# Experiences of living with funnel chest prior to corrective surgery: An interview study

**DOI:** 10.1371/journal.pone.0304968

**Published:** 2024-07-12

**Authors:** Louise Norlander, Agneta Anderzén-Carlsson, Mårten Vidlund, Ann-Sofie Sundqvist

**Affiliations:** 1 Department of Cardiothoracic and Vascular Surgery, Faculty of Medicine and Health, Örebro University, Örebro, Sweden; 2 Department of Perioperative Medicine and Intensive Care, Karolinska University Hospital, Stockholm, Sweden; 3 University Health Care Research Centre, Faculty of Medicine and Health, Örebro University, Örebro, Sweden; Sapienza University of Rome: Universita degli Studi di Roma La Sapienza, ITALY

## Abstract

**Objectives:**

Pectus excavatum, or funnel chest, causes both physical and psychosocial issues, affecting health-related quality of life. However, the literature on how funnel chest affects daily living prior to corrective surgery is sparse. Therefore, the study aimed to describe the experiences of living with funnel chest prior to correctional surgery.

**Materials and methods:**

The study had a qualitative exploratory design. Consecutive sampling was applied in which all individuals from a single cardiothoracic department scheduled for the minimally invasive repair of pectus excavatum were asked to participate. Nineteen participants, 17 men and two women, participated in the study. Individual telephone interviews were conducted from February 2020 until April 2021. The interviews were analyzed with qualitative content analysis using an inductive approach.

**Results:**

The overall theme “To have or not to have a cavity in my chest, it could make a difference” was interpreted as the latent meaning of the participants’ experiences. The theme included two subthemes with three categories each. The subtheme “The funnel chest puts a weight on my shoulders” describes the heavy burden the funnel chest places on the participants. The second subtheme, “This is me, but I want to change my future”, describes that participants see the funnel chest as a part of themselves; nevertheless, they look forward to surgery and a life without it.

**Conclusion:**

The results emphasize the heavy burden funnel chest causes and the great limitations it places on the individual. It also highlights the importance of surgery and the hope for a better future for individuals with funnel chest.

## Introduction

Pectus excavatum, or funnel chest, accounts for 90% of all thoracic deformities, with an incidence of 1 in 400 [1]. The deformity is a predominant rightward depression of the sternum and adjacent costal cartilage. Funnel chest is aggravated by growth spurts during childhood, reaches its full manifestation during puberty and thereafter stabilizes [2]. The intrathoracic volume often decreases, and the heart may be displaced from its original position. This causes many of the cardiopulmonary symptoms that individuals experience, such as breathing difficulties, palpitations and decreased exercise capacity [3]. Previous studies have reported that individuals with funnel chest have a decreased health-related quality of life compared to healthy individuals [4, 5]. This is due to the physical, mental and psychosocial impairments the majority experience. Low self-esteem, self-confidence and avoidance of social activities are commonly reported among individuals with funnel chest due to their disfigurement [6].

The gold standard of correctional surgery for funnel chest is the minimally invasive repair, which through the years has been modified and refined with improved outcomes [7, 8]. Indications for surgery are broad, including both physical and psychosocial issues related to the deformity. The deformity’s severity and related objective physical assessments are not always associated with the impairment caused by funnel chest; thus, even those with mild deformity could benefit from surgery [9]. The literature on how funnel chest affects daily living prior to correctional surgery is sparse. Such knowledge is essential for understanding individual reasons for surgery. Although questionnaire-based reports on health-related quality of life have previously been reported in individuals with funnel chest [4–7, 10], questionnaires do not allow participants to voice their experiences in their own words. Therefore, this study aimed to describe the experiences of living with funnel chest prior to correctional surgery.

## Materials and methods

This descriptive study had a qualitative explorative design. Consecutive sampling was applied to enroll individuals accepted for the minimally invasive repair of pectus excavatum in a cardiothoracic surgery department, with a catchment area including the south and middle of Sweden. Eligible participants were approached first by mail followed by a telephone call. A total of 27 individuals were invited; of these, 19 accepted invitation, five rejected participation, and three received postponed surgeries due to the COVID-19 pandemic and were therefore excluded. Participant demographics (Table 1) were collected with a study-specific questionnaire that participants filled out online after the interview.

**Table 1:** Participants’ characteristics.

N	19
Sex	
Male	17 (89.5)
Female	2 (10.5)
Age, mean ± SD (min–max)	22 ± 6.6 (14–36)
Education	
Compulsory	4 (21.0)
Upper secondary	9 (47.4)
University	1 (5.3)
Ongoing compulsory	1 (5.3)
Missing	4 (21.0)
Occupation	
Employed	8 (42.1)
Student	6 (31.6)
Job applicant	1 (5.3)
Missing	4 (21.0)

Data are given as n (%) unless otherwise noted

**Fig 1:**
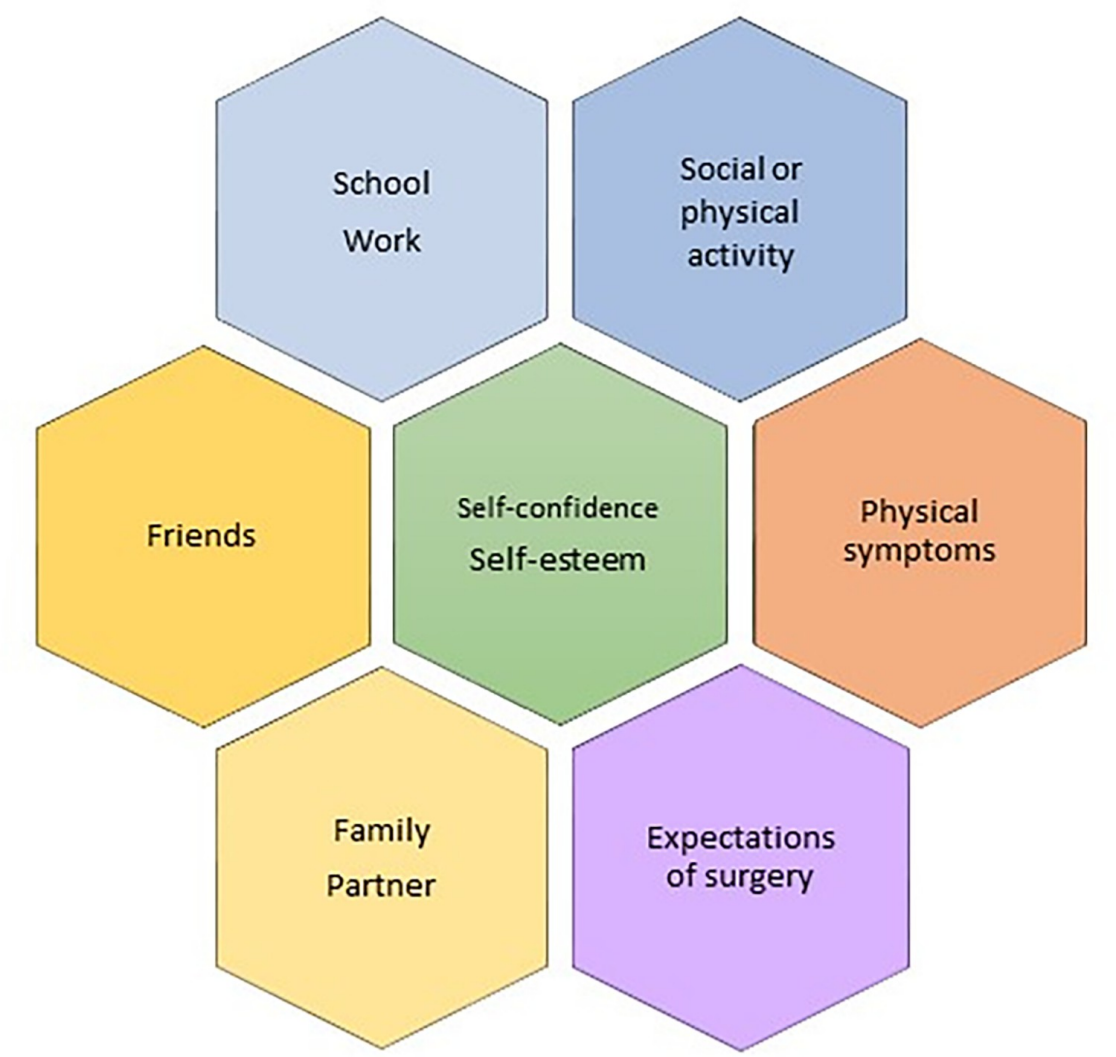
Overview of the topic guide.

Data were collected between February 25^th^ 2020 and April 30^th^ 2021 through individual telephone interviews that took place some days in advance of surgery. Telephone interviews were chosen due to the wide geographical residential area of the participants. Participants were asked to be situated in a calm environment where they felt safe to share their experiences. One participant had a parent present during the interview, and the remaining interviews were held one-on-one between the interviewer and the participant. The first author conducted the interviews. Interviews were semistructured with the opening question “How is your daily life affected by your funnel chest?” A topic guide was used to cover important aspects of life (see Fig 1). Interviews lasted between 15 and 74 minutes (mean 32 minutes), and they were audio recorded and later transcribed verbatim by a professional transcriber. No repeated interviews were carried out, and transcripts were not returned to participants for validation.

Data analysis was conducted using qualitative content analysis [11] with an inductive approach. The transcribed interviews were read thoroughly several times. Meaning units, that is, words or phrases with the same manifest meaning, were identified, condensed and labeled with a code by the first author. All authors categorized codes based on similarities and differences. In the iterative process, all authors compared the analyses with each other and grouped categories into subthemes based on their latent meanings. The overall theme was interpreted as the underlying meaning of the codes, categories and subthemes. All authors discussed differences and congruencies until consensus was reached. NVivo 14 software was used to manage the data. The consolidated criteria for reporting qualitative research checklist [12] for reporting of qualitative studies was used.

The study was conducted in accordance with the Declaration of Helsinki [13] and approved by the Regional Research Ethics Board Uppsala, Sweden (2018/365) and the Swedish Ethical Review Authority (2019–01177). Written informed consent was obtained from all participants. For participants <15 years old, additional written informed consent was obtained from parents or guardians. Participants were guaranteed confidentiality, and quotations from the interviews were identified only by numbers and not by name.

## Results

The analyses identified one overall theme, and two subthemes containing three categories each (see Table 2). The overall theme “To have, or not to have, a cavity in my chest–it could make a difference” was interpreted as the central meaning of how it feels to live with funnel chest and that corrective surgery could imply a change in the person’s everyday life. The theme covers the latent content of the subthemes, “The funnel chest puts a weight on my shoulders” and “Life must not be affected by my funnel chest”, embraces the categories, and is divided by negative and hopeful experiences of living with funnel chest prior to surgery. Participants experienced the content of both subthemes simultaneously, although the weight of their experiences had a negative focus.

**Table 2:** Overview of the categories, subthemes and themes.

Category	Subtheme	Theme
I feel different	The funnel chest puts a weight on my shoulders	To have or not have a cavity in my chest– it could make a difference
The funnel chest limits my everyday life		
I am emotionally affected by my different looks		
Learning about funnel chest was essential to me	This is me, but I want to change my future	
The funnel chest is a part of me		
There is hope for a better life		

### The funnel chest puts a weight on my shoulders

This subtheme describes the heavy burden funnel chest presents to participants. It includes categories with expressions of feeling different and the limitations that funnel chest places on everyday life. Moreover, this subtheme also covers the emotional consequences that funnel chest has caused.

#### I feel different

Most participants expressed that they felt different due to their chest and that they were alone with their feelings about it, feelings no one else could understand. The majority described that they did not want to show their chest to anyone, as it made them uncomfortable, and therefore, they hid it. They described that the appearance of their chest caused people to stare, wonder and make comments. Some participants stated that they felt exhausted by all questions they received and that it was difficult to talk about their funnel chest even with family members and close friends. They said they wanted to be like everyone else in the crowd, as stated by one of the participants, *“I just want to be normal*. *Let’s start there”* (interview 7).

#### The funnel chest limits my everyday life

The restrictions of everyday activities the funnel chest caused were expressed by the participants as limiting their lives. The majority felt that the funnel chest physically restrained them, some explained not being able to keep up with peers at sports, and others said that fainting forced them to stop doing what they loved. However, some stated that their funnel chest did not cause any physical limitations.

Participants also expressed how they suffered from the social impairments their funnel chest caused. Being in situations in which they anticipated taking off their shirt made many of the participants uncomfortable, i.e., sunbathing at the beach or showering after physical education class at school. Excuses, either to avoid showing their bare chest or to completely escape these situations, were common.

Participants described how they had adapted their behavior and created strategies to hide their deformity in order not to draw attention to themselves. They explained how they showered strategically when showering in public places, either first or last, or by changing clothes facing the wall. Others hid their chests with their arms. As one participant described, *“I try to hide by covering it* [the funnel chest] *with my arms*. *It’s kind a like*, *I put my hand over my shoulder*, *crossing and pretending to scratch my shoulder or something”* (interview 9). Clothes were also chosen with consideration of the funnel chest, and shirts that were tight or had a cut that was too low that exposed the shape of their chest made participants feel insecure and uncomfortable. In contrast, others expressed that clothes could make them feel safe and secure since the clothes made it possible to hide the deformity.

However, some explained that changing clothes with others in connection with youth sports was too difficult, forcing participants to quit the activity even though they wanted to continue practicing. The overall feeling participants experienced was that their whole lives felt limited due to their funnel chest: *“…it’s affecting not just myself*, *but my whole family*” (interview 14).

#### I am emotionally affected by my different looks

The funnel chest made the participants feel miserable. They also expressed feelings of shame, along with decreased self-confidence, self-esteem and self-image because of their appearance. The funnel chest made them withdraw from their social life, and the negative feelings that were expressed toward their deformity were clear. Someone explained that they hated their funnel chest. One participant described having had suicidal thoughts due to all the problems the deformity had caused: *“Like*, *it’s not worth living if you’re gonna hide yourself all the time*.*”–*Interview 19

Many also described that the funnel chest could be a barrier to intimate situations with others and that initiating new intimate relationships could be difficult.

*“I don’t know how long it took before I*, *was like completely open about it in front of my girlfriend and so… It took a couple of months at least*.*”–*Interview 11

### This is me, but I want to change my future

This subtheme contains categories that describes that although participants had felt they were different for a long time, many expressed relief when they received a diagnosis and gained more knowledge about their funnel chest. Moreover, despite the burden the deformity caused the participants, they regarded the funnel chest as a part of themselves, which they had been forced to live with. They were, therefore, longing for a life without it.

#### Learning about funnel chest was essential to me

Participants described how they usually noted that something was different about their chest from a young age or when entering puberty. Some had family members or schoolmates with the same deformity, while others gained knowledge of their funnel chest through social media or television. Many felt it was a relief to be offered an explanation for their different looks and that they were not alone in having this deformity.

*“I would say it was a huge relief*, *to like…* [realize] *that you are not alone with it… It’s funny*, *I actually understood what it was and the cause of it…”*–Interview 3

Social media and television also provided some of the participants with knowledge that their funnel chest could be corrected. However, when getting in contact with the health care system, the participants’ experiences differed. Some received accurate information about their deformity and surgical possibilities right away, while others experienced the opposite with flippancy and ignorance among health care professionals.

#### The funnel chest is a part of me

Although participants were not comfortable with their chest, they described how they had been forced to live with it. Growing older changed many participants’ feelings about their funnel chest. Some participants thought about their funnel chest more frequently when they were younger, while others had increased issues as they grew older. Likewise, there were different expressions of how they had come to live with their funnel chest. Some expressed that they could not be bothered to care about it, others that they had accepted it, and a few that they had just given up.

However, many felt that they had support from those close to them if they requested it. They felt accepted for who they were among family and close friends. A few stated that they felt no problem in looking different and expressed that they felt no shame for their chest and that it did not affect either their self-confidence or their self-image. One of the participants described it as follows: *“I usually don’t think about it that much and I’m not bothered of that people might stare and so… I can tell them what it is*.*”*–Interview 10. Individuals who experienced no problems with the appearance of their chest, primarily went through surgery due to physical problems.

#### There is hope for a better life

Participants expressed hope that surgery could ease their lives, both physically and psychosocially. The participants hoped that it would become easier to show their chest in public, that their self-confidence would increase and that their physical limitations would be reduced. Participants were excited about the upcoming surgery; however, many also felt anxious, especially about postoperative pain.

*“Yes*, *it’s two sided*. *It’s gonna feel so good*, *and it’s gonna be nice having it done and to go home and start life again*. *But at the same time it feels like it’s gonna be a couple of difficult days at the hospital*, *or rather*, *it will be*.*”*–Interview 1

Participants reflected on how surgery earlier in life would have affected them and felt sad for not being given the opportunity at a younger age. Some also emphasized the need for more widespread knowledge about the possibility of corrective surgery for funnel chest so that no one else would need to live a life as miserable as they had without knowing something could be done about it.

## Discussion

This study is the first to describe the experiences of living with funnel chest prior to corrective surgery as narrated by individuals with funnel chest themselves. The overall theme “To have, or not to have, a cavity in my chest–it could make a difference in my everyday life” shows that living with funnel chest is often a heavy burden both physically and psychosocially. Participants have been forced to learn to live with their deformity and manage life around it. Therefore, they looked forward to correctional surgery and hoped for a less burdensome life afterward.

The present results deepen previous understandings about individuals with funnel chest undergoing corrective surgery, which is mainly based on questionnaires with predetermined response options. Alaca and Yuksel [6] reported that patients with funnel chest experienced shortness of breath and chest pain more frequently than age-matched healthy controls. The same study stated that the patients also had higher levels of anxiety and social avoidance compared to controls. This could be further explained by our findings where participants described feeling miserable, along with quitting activities and avoiding situations where they felt restrained either physically or by the discomfort of having to show their chest, among others. The discomfort many experienced when exposing their funnel chest is probably a combination of many causes. Our results indicate that it is exhausting when people stare, ask questions and make comments that affect both the self-confidence and self-esteem of those with funnel chest and cause them to feel ashamed. This is similar to what Roberts et al. [7] found in patients prior to the minimally invasive repair and relates to the feelings of not being “normal”.

It has previously been described that individuals with chest wall deformity have skewed body images and perceptions of their physical appearance [9]. The present results provide further knowledge of how it is to live with funnel chest and that it is not solely a matter of body image and self-perception. However, during adolescence and early adulthood, body esteem (“the degree of positivity with which individuals regard the various parts of their body and the appearance of those parts” [14]) is suggested to interfere with individual development, as it is a time of rapid changes to both body and mind [15]. It could thus be argued that adolescents with funnel chest are at risk of having negative identity development due to the impairments the deformity brings. It is therefore important to follow up these children and adolescents and consider surgery when needed. Yet, further research about outcomes and comparisons of pre- and postoperative changes is also needed to fully evaluate the impact of surgical correction.

Previous research has focused on funnel chest and health-related quality of life by the use of self-report questionnaires. The Pectus Excavatum Evaluation Questionnaire (PEEQ) [16], Nuss Questionnaire modified for Adults (NQ-mA) [17], and Single Step Questionnaire (SSQ) [17] are the most frequently used tools, along with several modified [18–21] and translated [22–25] versions. The questionnaires include items regarding teasing, social distancing, self-confidence, shortness of breath and chest pain. Both the PEEQ and NQ-mA include items that ask if the condition makes respondents feel bad about themselves. However, the results of our study show that there are further dimensions to understanding how individuals with funnel chest feel, as participants described darker feelings and even suicidal thoughts. This result is especially important to keep in mind if the questionnaires are used in clinical praxis, as part of the decision-making process of whether to have surgery or nor, or in seeking psychological support.

Our results also showed that participants wished to have had surgery at a younger age. Some also experienced ignorance among health care professionals, suggesting that the knowledge of funnel chest and the possibility of surgical correction needs to increase, especially in primary health care and school health care. Better knowledge regarding funnel chest and its treatment options could possibly make more children and adolescents aware of their deformity earlier in life, thereby providing them and their families with accurate information and help to deal with eventual problems and treatment options. This could in turn lead to increased health for individuals with funnel chest.

To ensure trustworthiness in qualitative studies, there must be enough data to cover variations in experiences [26]. The final number of included participants in our study was considered sufficient in relation to the study’s aim, heterogeneity of sample, quality of data and method of analysis [27] and is believed to reflect the true experiences of the participants [26].

The authors’ preunderstandings of the subject may have influenced the study. NN (blinded for review) is a senior consultant cardiothoracic surgeon, with vast experience with the minimally invasive repair. NN (blinded for review) and NN (blinded for review) have extensive clinical experience in caring for patients with funnel chest as experienced registered nurse anesthetists at a cardiothoracic surgical department. NN (blinded for review) and NN (blinded for review) are experienced qualitative researchers contributing methodological knowledge. All these preunderstandings were considered during the planning and execution of the study and analysis. Furthermore, the topic guide, audio recordings of the interviews, and the separate and joint analyses made by all of the authors reduce the risk of author bias in the study. This also enhances the trustworthiness of the study.

## Conclusion

This is the first study to describe the lived experiences of funnel chest prior to the minimally invasive repair of pectus excavatum. Living with a funnel chest affects these individuals’ everyday lives both physically and psychosocially. The results emphasize the importance of surgery and imply the possible solution that the surgery could make their lives easier, thus increasing their health-related quality of life. Mental health and psychosocial support should be considered in school health care and primary health care, making individuals with funnel chest feel supported and thereby promoting their health.
